# Acquired Hemophilia A in an advanced age patient of hispanic origin: a case report

**DOI:** 10.1186/s13104-017-2767-6

**Published:** 2017-09-04

**Authors:** Nalyssa I. Rivera Cora, Freddie Irizarry Delgado, Santa M. Merle Ramírez, Jorge Vera Quiñones

**Affiliations:** 1 Family Medicine Residency Program, Bella Vista Hospital, Hwy 349 km 2.7, Cerro-Las Mesas, Mayaguez, PR 00681 USA; 2 University of Medicine and Health Sciences, 460 W 34th St., 4th Floor, New York, NY 10001 USA; 3 Western Hematology-Oncology Cancer Center, Cond. La Palma 1H, Calle Peral 14 N, Mayagüez, PR 00680 USA; 4 42 Castillo Street, Ponce, PR 00730-3741 USA

**Keywords:** Autoantibodies, Factor VIII, Acquired hemophilia, Bleeding, Hemostasis

## Abstract

**Background:**

Acquired Hemophilia A (AHA) is a rare hematological disorder that exhibits an incidence of approximately 1.5 cases per million patients a year. It is characterized by the development of autoantibodies against circulating Factor VIII coagulation proteins which, in turn, which in turn lead to potentially life-threatening hemorrhagic episodes. The incidence of AHA increases with age; with 80% of the affected patient population encompassing men and women that are 65 years or older. Some of the challenges that are highlighted in managing this disorder relate to the delayed diagnosis of this condition due to the rarity of the latter, the difficulty in establishing reliable hemostasis, and the secondary complications that are found when using immunosuppressive and hemostatic treatments in tandem with the elderly population afflicted with this disease.

**Case Presentation:**

A 90-year-old female of Hispanic origin presented with a 2-week history of generalized weakness, dizziness, shortness of breath and extensive purpuric formations that involved the left arm towards the lateral aspect of the thorax with the inclusion of a small right lateral neck hematoma formation. Upon initial laboratory screening, a hemoglobin level of 7.9, a hematocrit level of 21.9 and a PTT value of 70.9 were discovered. Despite conventional hemostatic treatment approaches, the patient did not show marked improvement of the laboratory values. Ongoing specialized laboratory reports, combined with the clinical presentation of the patient, led to the diagnosis of Acquired Hemophilia A. Treatment with recombinant porcine Factor VIII was initiated, which led to rapid improvement of clinical symptoms and laboratory values. The patient was discharged with current treatment plan and emergent follow/up with a hematologist was scheduled.

**Conclusion:**

Acquired Hemophilia A is an elusive bleeding disorder that has been seldom encountered in the demographics of Puerto Rico. The prompt detection of this diagnosis based on the clinical presentation alone is paramount to prevent the occurrence of grave hemorrhagic episodes. General knowledge and awareness of the treatment options available is key to ameliorate the prognosis of this ailment.

## Background

Acquired Hemophilia A is an uncommon autoimmune disorder that encompasses the encounter of autoantibodies directed toward inhibition of Factor VIII activity which serves as a cofactor for von Willebrand proteins, activated factor IX and phospholipids that harbor a negative charge [1–3]. The inhibition of this subject factor enables spontaneous bleeding episodes that can be life threatening [2]. With as many as 0.045 cases/million/year in children under 16 years of age and 14.7 cases/million/year for adults over the age of 65, there is a clear trend for this acquired disorder to be present in the elderly population [2–5]. The condition is characterized to harbor an association with an underlying medical condition in half of the reported cases such as chronic disorders (rheumatoid arthritis, inflammatory bowel disease, chronic obstructive pulmonary disease), malignancies and/or drug reactions [3]. The remainder of the reported cases are said to be idiopathic in origin [3].

This is a case-report of a Hispanic woman of advanced age who exhibited extensive purpuric lesions after a series of traumatic events and symptomatic anemia which, after laboratory confirmation, led to a diagnosis of Acquired Hemophilia A.

## Case presentation

**Fig. 1:**
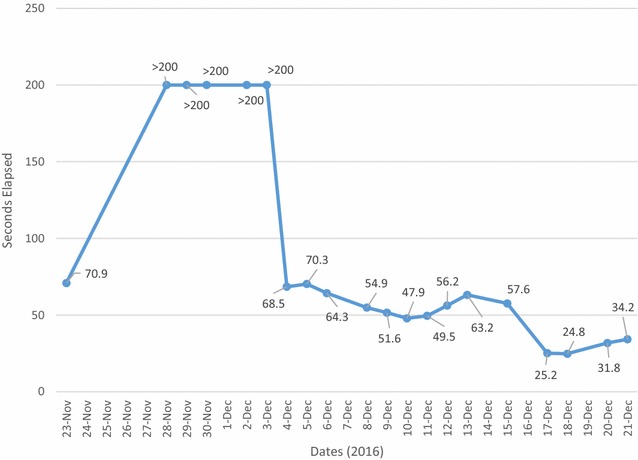
PTT (Dade) levels during inpatient stay. Description: PTT (Dade) Levels of the Patient during Hospital Stay demonstrate the effect of conservative hemostatic management of the patient vs. recombinant porcine factor VIII administration

**Fig. 2:**
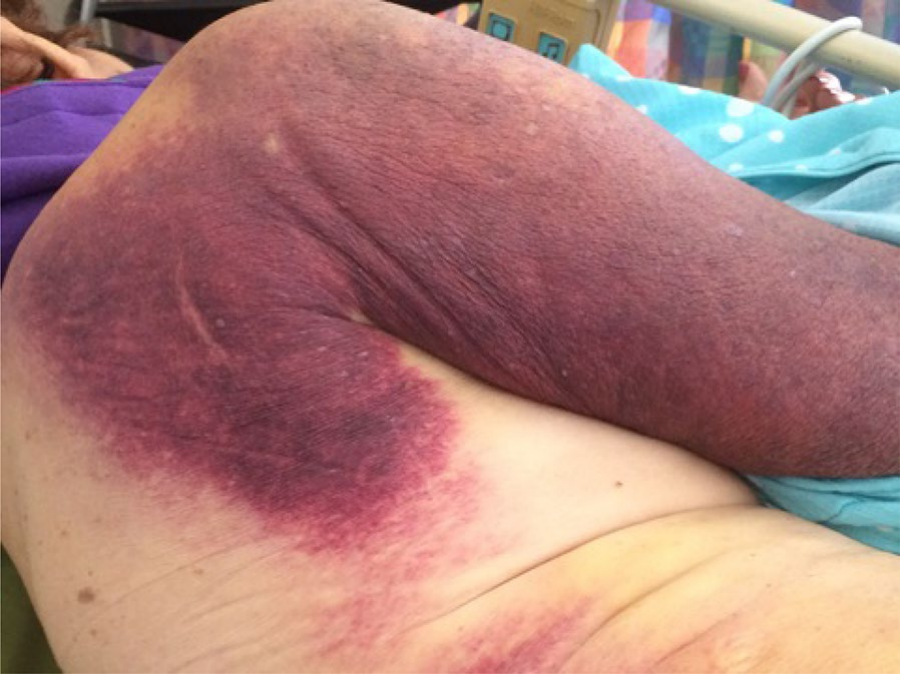
Extensive hematoma formation of the right shoulder with inclusion of the* right upper arm*

**Fig. 3:**
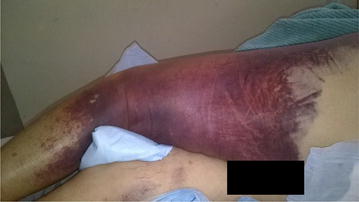
Hematoma of the Left thigh, *highlighting* the appearance of spontaneous subcutaneous hemorrhagic instances that is specific for AHA

A 90-year-old Hispanic woman presented with a 2-week history of symptomatic anemia and comprehensive purpuric lesions that extended from the left arm towards the lateral aspect of the thorax and a large hematoma on the left thigh. She had a past medical history of multiple falls, Alzheimer’s dementia, hypothyroidism, stage 3 chronic kidney disease and an uncorrected compression lumbar fracture (L3). Her surgical history was positive for a total abdominal hysterectomy, cholecystectomy, bilateral wrist surgery, left shoulder surgery and right knee surgery. Apart from maintenance pharmacological therapies, the only recent medication change was Duloxetine 60 mg OD, starting 4 days prior. Laboratory work-up was remarkable for a Hemoglobin level of 7.3 g/dL (Normal = 12.0, 16.0), a hematocrit percentage of 21.9% (Normal = 36.0–48.0%) and a PTT value of 70.9 > 200 s (Normal = 23.5, 30.3 s) (Fig. 1). Patient was admitted with the initial diagnosis of acute symptomatic anemia and GI consult was brought on board to determine potential sources of bleeding with Hematology/Oncology consult also being requested to explore alternative hematological diagnoses not considered initially. Management of the present condition was oriented towards conservation of hemostasis, administering PRBC, FFP and cryoprecipitate transfusions during acute exacerbations of anemia until a more definitive diagnosis was drawn upon. On day 10 of admission, Hematology/Oncology service determined the tentative diagnosis of Chronic DIC, due to the increased D-dimer of 4.89 (Normal = 0.00, 0.59) and an elevated FDP of 5.59 (Normal = 0.00, 0.59), and was given a total of 50 g of aminocaproic acid IV and started on Methylprednisolone 80 mg IV q8 h. Stool Occult Blood test was negative, subtracting diagnostic importance that suggests a lower gastrointestinal bleed. Urinalysis demonstrated no hematuria, which attenuated the relevance of the urinary tract as a source of the blood loss. Abdominopelvic CT scan without contrast demonstrated a poorly defined hypodense neoplastic process in the liver that measures approximately 4.5 cm; however due to persistently low hemoglobin levels, a liver biopsy was not attempted. Cardiac telemetry showed normal sinus rhythm; however, the latter did not offer diagnostic value. Anterior–Posterior Chest X-Ray did not contribute to the diagnosis. Left extremity Venous Doppler showed normal flow of the major vessels, withdrawing some diagnostic support from the left thigh hematoma as a local cause for the symptomatic anemia and ruling out deep venous thromboses. MRI of the right hip without contrast elucidated right gluteal and left thigh intramuscular hematomas, adding empirical evidence towards a chronic bleeding disorder related to the immune system. Physical examination supported the previous conclusions, showing extensive hematoma of the right shoulder (Fig. 2) and left thigh (Fig. 3). Due to the lack of clinical improvement, despite aggressive hemostatic measures (30 units of PRBC’s, 19 units of FFP and 10 units of cryoprecipitate were expended during inpatient stay), Hematology/Oncology consultation suggested the differential diagnosis of multiple myeloma versus Acquired Hemophilia A. Serum Protein Electrophoresis, Factor VIII activity assay and a Factor VIII inhibition profile was ordered to determine which of these conditions was prevalent on the patient. There was a faint IgG monoclonal band on electrophoresis, but not sufficient to diagnose a monoclonal gammopathy. The factor VIII activity assay showed less than 11% of activity, adding weight to the diagnosis of Acquired Hemophilia. The latter was further supported by a positive factor VIII inhibitory screen and the presence of Bethesda Units (BU) with a value of 76 (factor VIII-IgG complexes). Upon confirmation of the chief diagnosis, recombinant porcine factor VIII (rpFVIII) in conjunction with Methylprednisolone 500 mg IV and Cyclophosphamide 500 mg IV was given to the patient (12,000 units q6 h. IV 4× doses) which demonstrated remarkable improvement in Factor VIII activity (final measure read at 335% above baseline values). This is, in part, due to the overwhelming of the present autoantibodies with exogenous factor VIII with tandem immune suppression, which eventually decreased the acquired immune response to this clotting protein. As a follow-up to rule out underlying autoimmune disorders, titers for ANA, dsDNA, SS-A, SS-B, RNP, Scl-70, Smith, Ribosomal P, TPO, C3, C4 and RF autoantibodies were ordered and found to be negative for their respective conditions. Upon relative normalization of adjusted laboratory values in concert with the chronic ailments that the patient possesses, the latter was discharged with home care planning and subsequent hematology/oncology consult to follow up on the remission of the acquired autoimmune condition. The inpatient treatment strategies and acute management were in accordance with the wishes of family member in prolonging a stable health status for the patient, insofar no attempts were made to resuscitate the patient if the latter were to enter cardiopulmonary arrest.

## Discussion

Acquired Hemophilia A arises from the development of autoantibodies that are specific to the IgG heavy chain sub-classes IgG_1_ and IgG_4_ [1, 2, 4, 6]. These antibodies target to a specific carbon molecule (C2) of factor VIII, which bind to epithelial, von Willebrand factor-associated and activated platelet phosphatidylserine groups that regulate procoagulant activities [1, 2, 4]. The mitigation of Factor VIII cofactor functions in the intrinsic coagulation pathway essentially hampers the latter from effectivity of hemostasis and promotes spontaneous hemorrhagic episodes in soft tissues that are not specific in localization and/or exogenous insults and are extremely life threatening [1, 2, 4, 6]. This clinical manifestation is in contrast with congenital hemophilia A, which commonly presents with hemorrhage in joint articulations, muscles and other soft tissues that are manageable with conservative treatment [6]. Approximately half of all occurrences of AHA are attributed to a secondary cause such as rheumatic ailments, pregnancy, myeloproliferative disorders and malignant neoplasia with the other half of incidences possessing idiopathic tendencies [3, 7]. Some of the rheumatic disorders associated with AHA are rheumatic arthritis, systemic lupus erythematosus and autoimmune hypo/hyperthyroidism [3, 7]. Furthermore, autoimmune diseases such as chronic lymphoproliferative leukemia and multiple myeloma encompass a close relationship with this type of hemophilia. Solid tumor carcinomas that stem from the lung, breast and pancreas have demonstrated a proven link with the acquisition of this unusual blood disorder [3].

To confirm the presence of AHA, the clinical indications must be exhibited in addition with the specialized laboratory testing such as the Factor VIII inhibitory assay and Bethesda Unit quantification assay [7]. Initially, the patients who are afflicted with this disease do not have a family history of hematological disorders or any previous instances where uncommon pathological bleeding has been a chief complaint in their medical history [3, 7, 8]. This patient met with the previous prerequisites. This is followed with the ruling out of anticoagulant medications as the culprit of the bleeding episodes. Heparin can manifest the episodes that are native for this hematologic anomaly and the patient was not on anticoagulation therapy of any kind before the bleeding episodes [6]. There is also the matter of the laboratory studies that determine the inclusion of AHA as the main diagnosis. Factor VIII activity in this patient was less than 11% with BU values highlighting 76 units (Bethesda Units represent the quantity of antibody that would disable roughly half of the factor VIII activity in each sample), thus paving the way to a definitive diagnosis of Acquired Hemophilia A.

Given the immediate need for curative treatment and its ready availability, recombinant porcine factor VIII (rpFVIII) supplementation combined with immunosuppressants was used to manage and stabilize the present condition of the patient effectively. Clinically, the utilization of this therapy has shown hemostatic efficacy of over 90% and low cross-reactivity between anti-human factor VIII antibodies and rpFVIII is registered in several clinical studies [3, 7, 9–14]. In deference to other types of treatment such as Rituximab (anti-CD20 antibody) and tandem application of immunosuppressants such as prednisone and cyclosporine, rpFVIII was readily available and allowed us to monitor the activity levels of Factor VIII with expediency [3, 7, 9, 10]. Furthermore, the implementation of rpVIII was supplemented with the addition of Methylprednisolone and Cyclophosphamide to suppress the autoimmune response that engendered the reactive antibodies that targeted FVIII. The extended hospital stay (almost 1 month of inpatient services) and the surmounting costs of the administered treatments and blood transfusions hindered the hospital staff from implementing a complete treatment protocol in this patient. As such, the patient was discharged as soon as blood coagulation panels and hemoglobin/hematocrit levels normalized with the addendum of home care assistance and treatment follow-up by a hematology specialist.

This is the first case of this nature that was diagnosed in our hospital and the first time that recombinant porcine Factor VIII was used as a treatment. It is important to note, however, some possibilities that can explain the diagnosis in this patient. One of these would be the presence of a potential neoplasia in the liver that could have given way to the appearance of AHA in this patient. However, due to the strikingly low hemoglobin/hematocrit levels of this patient, a liver biopsy was contraindicated to determine the malignant potential of this abnormal mass. Since it is possible that solid tumors enable the generation of an acquired immune response with certain blood products, specifically coagulation factors such as factor VIII, it is not excluded in our analysis. Another possibility would be the fact that the patient has a history of autoimmune hypothyroidism, which could also attribute to the emergence of AHA since there is a proven tendency of the development of autoimmunity.

## Conclusions

Acquired Hemophilia A presents itself to be an elusive autoimmune disease that has a small footprint in the demographic makeup of Puerto Rico. More to the point, only one case has been reported in our statewide healthcare system in which a different treatment approach and clinical profile was encountered. Some points need to be mentioned which would add to the special nature of this clinical case. The human factor that led to the delayed diagnosis of this condition is unavoidable since common diagnoses such as lower GI bleeding, anemia secondary to chronic disorders and the presence of leukemia need to be ruled out before reaching the uncommon diagnosis of AHA. Finally, the difficulty in implementing hemostasis with conventional treatment protocols in patients with this anomalous condition needs to be considered. Training hospitalists and family medicine specialists in recognizing the clinical appearance of this bleeding disorder is key in determining the swift resolution of this treatable autoimmune disease.

## Abbreviations

AHA: Acquired Hemophilia A
ANA: anti-nuclear antibody
BU: Bethesda Units
C3: complement 3
C4: complement 4
dsDNA: double-stranded deoxyribonucleoprotein acid
FFP: fresh frozen plasma
IgG: immunoglobulin G
MRI: magnetic resonance imaging
PTT: partial thromboplastin
PRBC: packed red blood cells
RBC: red blood cells
RF: rheumatoid factor
Ribosomal P: ribosomal protein
RNP: ribonucleoprotein
rpVIII: recombinant porcine factor VIII
Scl-70: scleroderma-70
SS-A: Sjögren’s-syndrome-related antigen A
SS-B: Sjögren’s-syndrome-related antigen B
TPO: thyroid peroxidase
